# An uncertainty estimate of the prevalence of stunting in national surveys: the need for better precision

**DOI:** 10.1186/s12889-020-09753-8

**Published:** 2020-11-01

**Authors:** Santu Ghosh, Nirupama Shivakumar, Sulagna Bandyopadhyay, Harshpal S. Sachdev, Anura V. Kurpad, Tinku Thomas

**Affiliations:** 1 Department of Biostatistics, St. John’s Medical College, St. John’s Academy of Health Sciences, Bangalore, 560034 India; 2 Division of Nutrition, St. John’s Research Institute, St. John’s Academy of Health Sciences, Bangalore, India; 3 Sitaram Bhartia Institute of Science and Research, New Delhi, India; 4 Department of Physiology, St. John’s Medical College, St. John’s National Academy of Health Sciences, Bangalore, India

**Keywords:** Demographic health survey (DHS), Height-for-age, National Family Health Survey-4 (NFHS-4), WHO multicentre growth reference study (MGRS), Stunting, Overdisperion, WHO child growth standard

## Abstract

**Background:**

Stunting is determined by using the World Health Organization (WHO) child growth standard which was developed using precise measurements. However, it is unlikely that large scale surveys maintain the same level of rigour and precision when measuring the height of children. The population measure of stunting in children is sensitive to over-dispersion, and the high prevalence of stunting observed in surveys in low and middle-income countries (LMIC) could partly be due to lower measurement precison.

**Objectives:**

To quantify the incongruence in the dispersion of height-for-age in national surveys of < 5 y children, in relation to the standard WHO Multicenter Growth Reference Study (MGRS), and propose a measure of uncertainty in population measures of stunting.

**Methods:**

An uncertainty factor was proposed and measured from the observed incongruence in dispersion of the height-for-age of < 5 y children in the MGRS against carefully matched populations from the Demographic Health Survey of 17 countries (‘test datasets’, based on the availability of data). This also allowed for the determination of uncertainty-corrected prevalence of stunting (height-for-age Z score < − 2) in < 5 y children.

**Results:**

The uncertainty factor was estimated for 17 LMICs. This ranged from 0.9 to 2.1 for Peru and Egypt respectively (reference value 1). As an explicit country example, the dispersion of height-for-age in the Indian National Family Health Survey-4 test dataset was 39% higher than the MGRS study, with an uncertainty factor of 1.39. From this, the uncertainty-adjusted Indian national stunting prevalence estimate reduced to 18.7% from the unadjusted estimate of 36.2%.

**Conclusions:**

This study proposes a robust statistical method to estimate uncertainty in stunting prevalence estimates due to incongruent dispersions of height measured in national surveys for children < 5 years in relation to the WHO height-for-age standard. The uncertainty is partly due to population heterogeneity, but also due to measurement precision, and calls for better quality in these measurements.

**Supplementary Information:**

The online version contains supplementary material available at 10.1186/s12889-020-09753-8.

## Background

Stunting is the most frequently used indicator for chronic undernutrition in children and in setting priorities for interventions based on the Sustainable Development Goals [1] or the Global Hunger Index [2]. Though, the prevalence of stunting in some of the Latin American countries like Brazil, Peru and Bolivia has markedly reduced in last three decades [3], it continues to be high in other Low and Middle Income Countries (LMIC), especially in South Asian and sub-Saharan African countries [4]. For example, India reported a national average of 36% in < 5 y children in National Family Health Survey (NFHS-4) [5] and 35% in 0–4 y children in the recent Comprehensive National Nutrition Survey (CNNS) [6]. This measure of chronic underunitrition is related to poor outcomes in health, cognitive development, educational and economic attainment later in life [7].

Stunting is defined as a deficit in height relative to a child’s age, that is, 2 standard deviations (SD) below the median height-for-age derived from the WHO (World Health Organization) child growth standards (hereon referred to as the WHO standard) [8]. This growth standard is based on the WHO Multicentre Growth Reference Study (MGRS) [8], of the anthropometric indices of children living in what were considered to be the ‘best case’ for socioeconomic circumstances and nutritional access [8]. In the MGRS, the very low (~ 3%) between-country variation of age-specific mean height, and a strong similarity in the mean and SD of linear growth from birth to 5 y in different country samples provided sufficient justification for pooling data across countries to obtain a global standard [8].

The population estimate of stunting is sensitive to the dispersion of the height distribution in the population survey to which the standard is applied [9]. Maintaining a desired level of precision in length or height measurement is a major challenge in any large scale survey. Low precision will cause over-dispersion and thus overestimate a dispersion-sensitive measure such as stunting [10]. Simulation exercises showed that even modest, random errors in height measurements can increase the measure of dispersion and the increase in error can exponentially increase the dispersion [10]. It was also clearly demonstrated that this overdisperson could inturn inflate estimates of standard deviation cut-off based undernutrition indicators. In addition to the measurement error induced over-dispersion [10–12], population heterogeinity due to biological variability, socioeconomic inequalities and adverse environmental exposure would independently contribute to higher dispersion compared to that of the standard population.

Prompted by the concerns on data quality and its impact on nutrition indicators, standard methods such as the Standardized Monitoring and Assessment of Relief and Transitions (SMART) [13] have been developed for quality assessment of anthropometric measures for large scale surveys, at the time of data collection, reporting and interpretation [10, 11]. However, there is no method that provides a quantitative measure of the potential measurement errors and a measure such as this could further be used re-estimate the prevalence of stunting, after accounting for an uncertainty factor. The aim of the study is to identify an estimate of uncertainty that can be obtained by comparing the dispersion of height-for-age in a sample of healthy children at any given age living in growth-favorable environments in the national survey, with the dispersion of the MGRS. This estimate of uncertainity can be used to correct for the incongruence in dispersion and obtain a rough estimate of the stunting in the absence of measurement error in height. Since the prevalence of stunting is a commonly used population level indicator to monitor and assess the effectiveness of public health and nutrition policy/programs, [14] it is worth quantifying an uncertainty factor to interpret these data and estimate a dispersion corrected prevalence.

In the current paper, we first explored the possibility of overdispersion in height for age measurement in NFHS-4 survey data. Then we proposed a statistical method to quantify overdispersion or uncertainty of the estimate along with a statistical test. A method of dispersion corrected prevalence estimation of stunting was proposed and was applied to DHS and NFHS-4 survey data sets.

## Methods

The first part of this study explicitly explored a specific national survey to identify over-dispersion in height-for-age as a potential source of uncertainty. For this exercise, the Indian NFHS-4 survey [5] conducted in 2015–16 was used, which provides a nation-wide detailed database on socioeconomic status and anthropometric measurements (237,136 valid height measurements) of < 5 y children (details of survey methods given in Additional file 1). The biologically implausible data was defined as height-for-age z-score (HAZ) > 6 or < − 6 of the sample mean and were cleaned as per the defined norms of WHO [15]. Normality of the HAZ distribution were examined for skewness and kurtosis [16]. The height-for-age data in < 5 y children from NFHS-4 [5] were classified into percentiles under each wealth quintile (computed based on the reported possessions and other household characteristics) and then compared to the WHO standard. Generalized Additive Models for Location, Scale and Shape (GAMLSS) [17] was used to derived smoothed percentiles across the quintiles.

In the second part of this study, the impact of over-dispersion of height-for-age on global country estimates of stunting was examined in the DHS survey data [18] of < 5 y children residing in LMIC (the method of data extraction method from DHS is provided in Online Additional Material 1).

### Statistical method for obtaining an uncertainty factor due to measurement error

Let *y*_*t*_ be the height-for-age t in a given healthy population with growth favourable environment and *y*_*t*_ is subjected to measurement error due to low precision. Let *Y*_*t*_ be the true measurement of the height-for-age t, then observed *y*_*t*_ can written as

$$ {y}_t={Y}_t+{e}_t;{Y}_t\sim N\left({\mu}_t,{\sigma}_t^2\right)\&{e}_t\sim N\left(0,{\sigma}_e^2\right) $$

where *e*_*t*_ is random measurement error possibly due to low precision in the measurement and the variance is assumed to be age invariant, as only a trivial difference was observed between that of children below 24 months and children above 24 months. Hence observed height (*y*_*t*_) will have normal distribution with mean *μ*_*t*_ and variance

$$ {\sigma}_t^2+{\sigma}_e^2={\sigma}_t^2\left(1+\frac{\sigma_e^2}{\sigma_t^2}\right)={\sigma}_t^2{\delta}_t^2. $$

For sake of simplicity, we assume ${\delta}_t^2$, the over-dispersion measure (uncertainty factor) to be age invariant [${\delta}_t^2={\delta}^2\in \left(0,\infty \right)\Big]$.

As the WHO growth standard is applicable globally, the true mean and variance of height of the healthy population assumed above is assumed to be exactly equal to the WHO standard height for the age t. Hence ${\mu}_t={\mu}_t^{WHO};{\sigma}_t={\sigma}_t^{WHO}$ and

$$ Z=\frac{y_t-{\mu}_t^{WHO}}{\sigma_t^{WHO}}\sim N\left(0,{\delta}^2\right) $$

which is the Z-score as per WHO standard height-for-age t. Let {*Z*_1_, *Z*_2_, …, *Z*_*n*_} be the HAZ for a sample of size n drawn from the healthy populaution identified above. An obvious estimate of *δ*^2^ would be

1 $$ {\hat{\delta}}^2=\frac{1}{n}\sum \limits_{i=1}^n{Z}_i^2 $$

The uncertainty factor *δ* lies between (0, ∞); *δ* = 1 indicates no over-dispersion; *δ* > 1 *or δ* < 1 indicates over-dispersion or under-dispersion against WHO standard height-for-age.

A *χ*^2^ statistical test (*H*_0_ : *δ* = 1 *vs*. *H*_1_ : *δ* ≠ 1), can also be performed in the sample to test for significant over-dispersion or under-dispersion. The details of testing procedures are provided in Additional file 2.

Finally, estimated *δ*, or the over-dispersion measure, can be used to adjust the Z-score of the survey data prior to deriving stunting as follows

2 $$ {Z}_j^{RS}=\frac{Z_j}{\hat{\delta}}\  for\  all\ j=1,2,\dots, m\left( survey\ size\right) $$

A child whose ${z}_j^{RS}<-2;j=1,2,..,m$ would be defined as stunted adjusted for dispersion by the survey tool.

For computing this uncertainity factor for any survey, the requirement is to identify a population that has a overdispersion compared to WHO MGRS, solely due to measurement error of height and not due to other biological variability, socioeconomic inequalities and adverse environmental exposure. A sample from such a population would be considered as a ‘test dataset’.

### The extraction of a ‘test dataset’ of healthy children with a favourable growth environment for comparison of dispersion with that of the WHO MGRS

A true ‘test dataset’ should be a sample from a healthy sub-population with a favourable growth environment for the given population. If not available, an attempt to obtain a suitable subsample could be made from the same survey data, identifying a healthy subset that represents the population of healthy children as per the standard definition. As WHO growth standard was developed on a sample of healthy children, the inclusion and exclusion criteria of MGRS [8] data set were used to indentify a subset from NFHS-4 survey that approximate desired healthy sample of children below 5 years. Hence, the test dataset was extracted by matching children by ‘locality’, ‘socio-economic status’, ‘mother’s education’, ‘non-smoking mothers’, ‘exclusive breast feeding for 4 months’, ‘partial breast feeding for 12 months’ with MGRS [8]. This test dataset (*n* = 3732) consisted of children in the richest wealth quintile of an urban locality, born with normal birthweight, breastfed till 4–23 months of age, and whose mothers were educated to graduate level and above. Outliers for height in this sample, below the 5th and above the 95th percentile of HAZ, as derived by the WHO standard, were excluded.

Several steps were taken to evaluate the validity of the above measure of uncertainty factor (δ). First, the variance in the height-for-age of the NFHS-4 test dataset was compared with other Indian studies [12–14]. Reports published between January 2004 and March 2019 (since MGRS completed in December 2003) in PubMed, identified with search terms: ((((Infant) OR Children) AND Height) OR Anthropo*) AND India, yielded 3 studies [19–21] on children aged 24–59 months. Single site studies on >5y children, clinical complications, and those reporting on children from middle or lower socio-economic background were excluded. Second, a sensitivity analysis was conducted with different choices of the matching variables on the prevalence of uncertainty adjusted stunting, since the test dataset was selected by matching socio-demographic characteristics of the Indian sub-sample of the MGRS [8]. Third, using the same criteria as described above to extract NFHS-4 test dataset, statistical matching was applied to earlier Indian NFHS-3 data [22], which was conducted between 2005 and 2006, and the estimated uncertainty factor then compared between the two successive Indian NFHS surveys, along with the calculated uncertainty-adjusted stunting prevalence.

### Estimation of the uncertainty factor in demographic health survey (DHS) data

In the second part of this analysis, the uncertainty factor was estimated for surveys from the 17 DHS countries that had socio-demographic data available to allow matching with the WHO MGRS data, and had a sufficient sample size for the estimation of the uncertainty factor (details of computation provided in Additional Figure 1). Standard method and measurement protocols were used to collect DHS survey data [23]. The unadjusted stunting prevalence in all the selected countries was estimated using the WHO standard [8]. The estimates of uncertainty-adjusted stunting were then calculated for those countries with sufficient evidence for *δ* being different from the Null value of 1, ie: ${\chi}_{0.05.;n}^2<{\chi}_{stat}^2<{\chi}_{0.95.;n}^2$.

All data analyses were performed using R-version 3.6.1 [24].

## Results

### Uncertainty factor for the Indian NFHS-4 survey

**Fig. 1:**
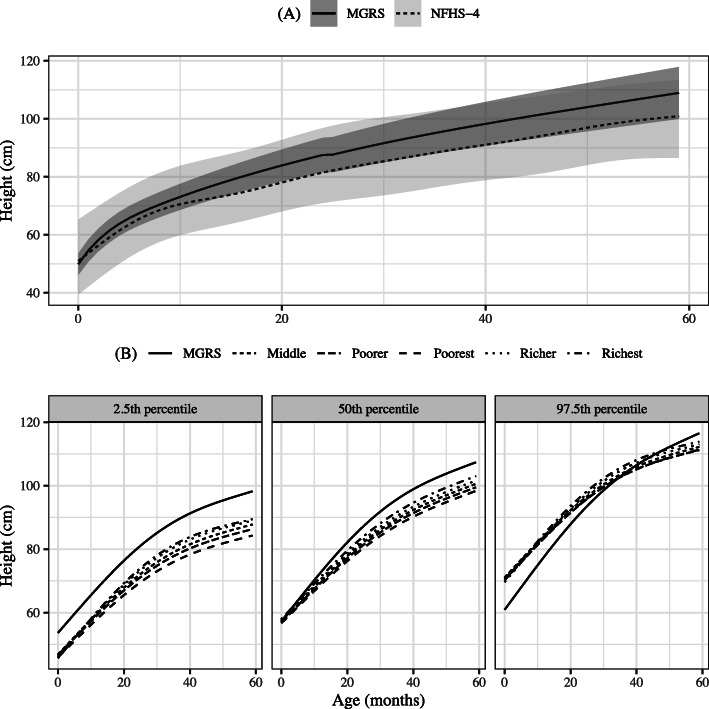
Distribution of MGRS and NFHS-4 height-for-age; **a** Median and 95% CI of height-for-age from MGRS and NFHS-4 (**b**) Height-for-age centiles of < 5 y children from MGRS and NFHS-4 in different wealth quintiles; NFHS-4 (*n* = 237,136), richest (*n* = 32,152), richer (*n* = 39,607), middle (*n* = 47,360), poorer (*n* = 55,916), poorest (*n* = 62,101). CI: Confidence Interval; MGRS: WHO Multicentre Growth Reference Study [6]; NFHS-4: National Family Health Survey-4 [4]

A consistent over-dispersion of the height-for-age distribution was observed in the NFHS-4 data when compared to the MGRS (Fig. 1a)**.** The height-for-age percentile curves (2.5th, 50th and 97.5th) constructed from NFHS-4 data, classified by wealth quintiles, are shown in comparison to the MGRS, in Fig. 1b. The 2.5th percentile curve (Fig. 1b) remained consistently lower and 97.5th percentile curve remained consistently higher upto ~ 3 years before it started showing little downward trend for NFHS-4 data in comparison to the same curve for the MGRS across age groups and wealth quintiles (Fig. 1b), indicating greater dispersion of NFHS-4 height. However, median shows gradually decreasing trend as age pogressess compared to MGRS.

The estimated value of the $\hat{\delta}$ (uncertainty factor) was 1.39 (95% CI: 1.36–1.43), which meant that the dispersion in height-for-age in the NFHS-4 test dataset was 39% higher (*P* < 0.001) than that observed in the MGRS data [8]. Using the uncertainty adjusted Z-score for the WHO standard, the prevalence of stunting could be as low as 18.7%, since the dispersion was much higher than MGRS**.** This value is substantially lower than the current estimated prevalence of 36.2% (calculated from the raw data of NFHS-4) using the unaltered WHO standard. The estimates of $\hat{\delta}$ for younger children (< 24 months) was 1.40 (95% CI: 1.36–1.45) and was comparable to that of children aged 24–59 montsh, $\hat{\delta}$ =1.38 (95% CI: 1.33–1.44).

**Fig. 2:**
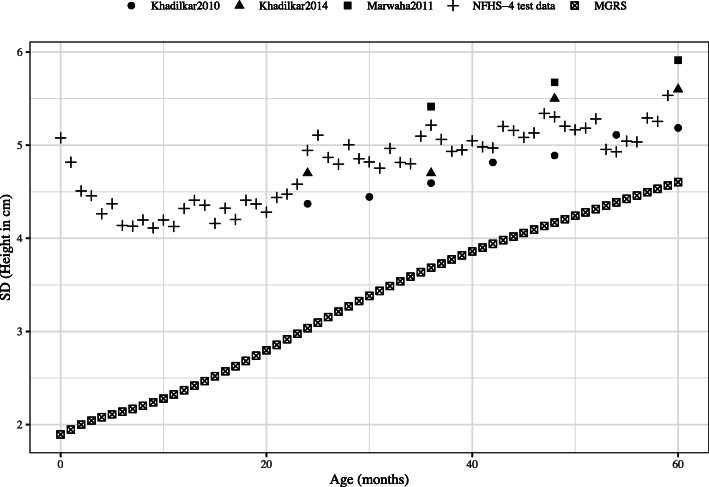
Comparison of height-for-age SD in MGRS with NFHS-4 test dataset and other Indian studies; Khadilkar 2010 (*n* = 1493), [13] Khadilkar 2014 (*n* = 494), [12] Marwaha 2011 (*n* = 106,843 children of 3–18 y), [14] NFHS-4 test data (*n* = 3732), [4] MGRS (*n* = 8440) [6]; SD: Standard deviation; MGRS: WHO Multicentre Growth Reference Study [6]; NFHS-4: National Family Health Survey-4 [4]

The dispersion of the height-for-age distribution in the test dataset of NFHS-4 was comparable to other nationally representative datasets but was much higher than that of the MGRS, which defines the WHO standard, as shown in Fig. 2. The sensitivity analysis of the determination of δ, with different choices of matching variables between NFHS-4 data and the Indian sub-sample of the MGRS [8] is reported in Table 1. Maternal education and wealth quintiles were sensitive, but to a small extent. The uncertainty factor varied from 1.39 to 1.43 when maternal education (up to graduate level and above) was replaced with education up to matriculation and above. When replacing the richest quintile with the top two quintiles of wealth, the uncertainty factor increased from from 1.39 to 1.50 (Table 1). These slight changes in uncertainty could result in small changes in the uncertainty adjusted prevalence of stunting.

**Table 1:** Sensitivity analysis of uncertainty factor based on socio-demographic characteristics

Parameters	N	δ
NFHS-4 test data set	3732	1.39
Wealth quintile: Richer/Richest	775	1.50
Locality: Rural	1638	1.38
Birth weight > 3 kg	2373	1.40
Breast feeding: 6–24 months	2973	1.40
Education: > 9 y	6826	1.43
Education: > 11 y	5523	1.42

NFHS-4: National Family Health Survey-4 (5) n: sample size of NFHS-4 test dataset with select values on matching variables from MGRS; δ: Uncertainty factor for over-dispersion of height-for-age observed in NFHS-4 test data set compared to the MGRS (WHO Multicentre Growth Reference Study) [8].

As an additional test of validity, a consistent over-dispersion of the height-for-age distribution was observed in the earlier NFHS-3 survey as well, when compared to WHO standard (Additional Figure 2A). Applying the same matching criteria (as performed for the NFHS-4 survey) to the NFHS-3 survey data to obtain a similar test dataset (Additional Figure 2B) of the healthiest children (*n* = 828), the *δ* (dispersion based uncertainty factor) was found to be 1.31 (95% CI: 1.24–1.38,), which was not different from the *δ* value estimated for the NFHS-4 survey (Additional Figure 3). The uncertainty adjusted prevalence of stunting obtained in the NFHS-3 survey was 32.3% (95% CI: 31.9–32.8), compared to 48% (95% CI: 47.6–48.5) when estimated by the unaltered WHO standard.

### Uncertainty factor in selected DHS country data

**Fig. 3:**
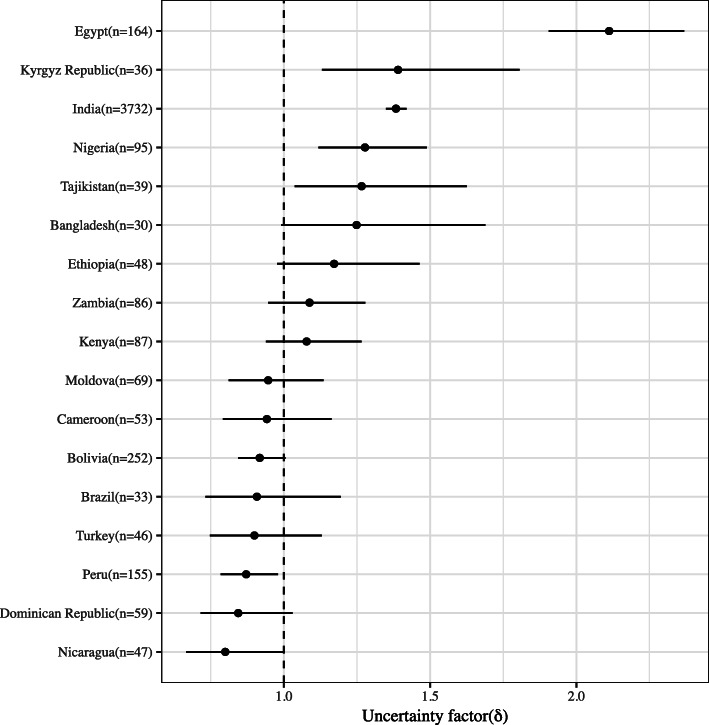
Estimated value of uncertainty factor for variance of height-for-age using DHS data in LMIC countries; DHS: Demographic Health Survey [11]; LMIC: low- and middle-income countries

The uncertainty factor *δ* could be calculated for 17 selected DHS countries, where data were available, and ranged from 0.80 (95% CI: 0.67–1.00) for Nicaragua to 2.11 (95% CI: 1.91–2.37) for Egypt (Fig. 3). The *δ* was lower than 1 for Latin American countries, with Peru having 13% lower dispersion for height-for-age in their test dataset (*δ* =0.87, 95% CI: 0.78–0.98). Most of the African and all Asian countries selected from the DHS had *δ* > 1 indicating over-dispersion. The *δ* value for India (1.39) was higher than most DHS countries, but lower than that of Egypt which had the highest estimate (*δ* = 2.1; 95% CI:1.9–2.4). As a result, the uncertainty adjustment lowered the stunting prevalence in Egypt to 3% compared to the 21% unadjusted stunting prevalence estimated with the WHO MGRS standard [8].

## Discussion

The debate about stunting as a primary growth problem, rather than an indicator of social deprivation, poverty and environment, and the consequent diversion for attention from the underlying causes has been pointed out before [25, 26]. This has led to debates about the application of global standards to Indian, and by extension, all LMIC children. In some instances, such as in India, these debates have focused on the potential genetic predisposition to short stature, with counterpoints linked to the high variation in height-for-age [25, 26]. In a population living under ideal conditions for child growth, ∼2.5% of the children would be stunted, where the HAZ would be normally distributed with a mean value of zero and SD of 1 [8]. In large scale surveys [27, 28], it is often observed that the SD of the HAZ distribution is > 1, suggesting that the prevalence of stunting (HAZ < -2) may be > 2.5% even in the healthiest subset of the population due to higher variablility in the anthrometric data in most of the large surveys [29]. This is problematic as the HAZ metric is based on 2.5th percentile position of the height-for-age and distribution in a standard healthy population as defined in the WHO MGRS [8].

As a first step, a quantitative approach to measure and correct for incongruencies in dispersion is required as stunting is a commonly used indicator to evaluate policy and programs and therefore it is worthwhile to dispersion corrected prevalence for more precise estimates of change or progress. The present study makes the first attempt to offer an appropriate, simple, but robust method to examine the uncertainty in the application of the WHO standard [8] in accordance to the variation observed in a relatively healthy population (test dataset of the NFHS-4 survey in this instance), and thereby offers a measure of an over-dispersion based uncertainty factor. The sensitivity analysis (Table 1) performed on the uncertainty factor $\hat{\delta}$ showed that matching criteria for the test dataset were stringent. Using this estimate of the factor to adjust the stunting prevalence estimates gives values that are, in some cases, far removed from the unadjusted prevalence estimate (for example, Egypt and India). Therefore, it is important to be cautious that the estimation of the uncertainty factor is indicative, but may not result in a ‘true, corrected’ stunting prevalence. There is simply no replacement for better quality and precision in measurements performed in national surveys, which can be achived with available guidance during conduct of large surveys [16].

However, for a survey with optimal measurement error with respect to MGRS study, a deviation of this factor from ‘1’ would indicate true population variation and imply the requirement for population adjustment in dispersion while using standard data. A true higher dispersion could occur due to biological and environmental reasons. These could be related to intergenerational and maternal diet linked epigenetics, parental height, intra-uterine growth restriction, and variation in gestational age at birth [30], or an unsanitary and polluted environment arising from poverty. The sampling method for the MGRS was careful to reduce this variation, by drawing samples from a well-defined affluent population in a single city of the selected country. However, since 85–90% of total genetic variation is found in a collection of individuals within country, multisite sampling within-country is probably a requirement for a true representation of the country population [31]. The study attempted to quantify over-dispersion in the distribution of height-for-age using a ‘best’ subset of the survey itself, and importantly, does not differentiate over-dispersion due to true variability or measurement error.

Thus, in the test dataset of the national surveys studied here, the over-dispersion of height-for-age could be first, due to random errors introduced by poor precision in the measurement of height, recall bias related age-ascertainment errors, terminal digit preferences, incorrect data recording and entry [10, 11]. Second, there is always the possibility that the true biological variance of the theoretically healthiest children could be higher than that of the MGRS. Third, the study design and sampling techniques followed in these large scale national surveys [5, 18] and the MGRS were different [8]. Fourth, secular trends in the variance of height may be operative. However, this was tested in Indian surveys, where a similar over-dispersion in height-for-age was observed in the earlier NFHS-3 (Additional Figure 2), as well as in other research studies in India (Fig. 2); thus it is unlikely that this was responsible for the over-dispersion observed in the later NFHS-4. The overdispersion was considered to be age invariant and this assumption was tested in the NFHS-4 data set, and the uncertainty estimate may vary by age if the measuremenr error varies by age, which appears unlikely.

A recent analysis of height distribution from 179 Demographic and Health Surveys in 64 low and middle-income countries, reported the mean SD of HAZ to be 1.68 (range: 1.65–2.11), indicating the possibility of greater uncertainty in these countries when using the WHO standard [29]. The same analysis showed that if all children were exposed to the same growth limiting factors, the mean height-for-age would decline without an increase in the SD [29]. This may explain the observed lower dispersion but higher prevalence of stunting in sub-saharan African and Latin American countries (Fig. 3).

Measuring the height or length of children is not trivial, defined precision is an important part of survey reporting [10, 16]. The reported precision of the height measurement in the MGRS was excellent, at < 1%, and procedures were adopted to maintain precision throughout the study keeping minimal inter-observer variability [8]. Rigorous training, periodic standardization sessions and assessments, frequent monitoring and regular equipment calibration were among the quality measures of the MGRS [8]. The DHS and the NFHS-4 do not report the precision of the length or height measurements that were made, although the measurements were made using standard instruments [5, 18]. Other cross-sectional studies from India, refered in the present analysis, have reported a precision for height measurement of < 1% at the beginning of the survey, [19–21] but it is not clear how precision was maintained throughout the survey period. A comparative analysis of the HAZ distributions for large scale population based surveys such as DHS and Multiple Indicator Cluster Surveys (MICS) showed greater SD of HAZ (1.82 and 1.80 respectively) compared to National Nutrition Surveys (NNS) (SD – 1.36), which followed the standardised SMART method to conduct the survey [11] suggesting the importance of quality assessment in athropometric measures. However, the uncertainty adjustement will not affect a trend analysis of stunting prevalence between NHFS-3 and NFHS-4 as the adjustment factor is very similar, but a comparison of the uncertainty adjusted prevalence estimates between different countries will be preferable given the adjustment factor is different by country. A height distribution analysis using Joint Malnutrition Estimates database (included data from 422 surveys) showed that SDs of HAZ progressively decreased with age, ranging from 1.59 in 6–11 months to 1.28 in 48–59 months, indicating the complexity of measuring length compared to height in children [16]. Higher variation could also be observed if inconsistent measurement techniques were followed; for instance, one versus two leg recumbent length measurement (0.02 to 2 cm difference) have shown a random variation which decreases with increasing age [32]. The diurnal variation in height measurement [26] and errors in maternal recall of birth dates [33] are other important aspects that could lead to imprecise measurements of height. A random error of 2 cm in height measurement can increase the SD of HAZ distribution to 1.17 which may in turn overestimate the prevalence of moderate and severe stunting by 3.5 and 2.2% respectively. The same analysis showed that every 0.1 SD reduction in over-dispersion can reduce the prevalence of stunting by 2% [10]. Further, the random errors associated with accurate age determination also have shown to impact over-dispersion of height-for-age distribution. This is critical when age is approximated using a calendar of local events and mother’s recall ability, in the absence of accurate birth records. An error of 3 months in a 12 month old child can increase the SD of height distribution up to 1.97 and the prevalence of severe stunting can be overestimated by 7.3% [10].

In addition, the purposive sampling and longitudinal study design (0–2 y), followed in the MGRS were not comparable to the cross-sectional sampling design of the DHS. The sampling variation with random sampling in a cross-sectional study design will always be higher than purposive sampling in a longitudinal study design. On the other hand, with a higher replication, the approximation of sampling distribution of any sample statistic is likely to be better. It is possible that having only 6 samples from 6 study centers in the MGRS study [8] may still be insufficient to accurately estimate the true sampling distribution of the sample variance of the population used to derive the growth standard. Effectively, the homogeneity across samples from different countries, or within a country, needs to be established with more replication, under the same conditions. Quantification of uncertainty due measurement error and a novel method of its correction in prevalence of stunting are the most important strengths of the study. A limitation was matching for partial number of inclusion and exclusion criteria (gestational age, history of perinatal morbidity and time of complementary feeding introduction were not available) with the MGRS Indian subsample [6] to extract the DHS test dataset. An additional limitation is the inability to differentiate true population heterogeneity from measurement imprecision in the test dataset.

## Conclusions

In conclusion, the uncertainty factor, derived by adjusting for over-dispersion from a test dataset (extracted from a national survey) in comparison to the dispersion in the WHO standard, provides a comprehensive approach that corrects for both precision errors in a large-scale survey and true biological variation of a population. Although, true population heterogeneity could not be differentiated from measurement imprecision in the test dataset, the aim here was to provide a level of uncertainty to be used while interpreting stunting prevalence estimates. A similar over-dispersion could occur with the measurement of weight as well and the present attempt needs to be expanded for other measures of undernutrition such as weight-for-age and weight-for-height. This analysis emphasises the critical need for maintaining precision in anthropometric measurements, especially in large surveys used to derive nutritional status indicators that in turn inform policy.

## Supplementary Information

**Additional file 1.** National Family Health Survey-4 (NFHS-4) methodology; This file provides details of the NFH 4 survey methodology

**Additional file 2.** Derivation of test statistic based on Z-score; Describes the derivation of test statistic over or under dispersion

**Additional file 3: Additional Figure 1.** Flow diagram of country selection from DHS data to calculate the prevalence of relative stunting; DHS: Demographic Health Survey

**Additional file 4: Additional Figure 2.** Distribution of height-for-age in NFHS-3 and MGRS

**Additional file 5: Additional Figure 3.** Distribution of height-for-age in NFHS-4 test data set and MGRS

## Data Availability

The datasets analysed during the current study are available in the DHS data repository, [https://www.dhsprogram.com/data/available-datasets.cfm].

## Abbreviations

CI: Confidence Interval
CNNS: Comprehensive National Nutrition Survey
DHS: Demographic Health Survey
GAMLSS: Generalized Additive Models for Location, Scale and Shape
HAZ: Height-for-age z-score
LMIC: Low and Middle Income Countries
MGRS: Multicentre Growth Reference Study
MICS: Multiple Indicator Cluster Surveys
NFHS-4: National Family Health Survey
NNS: National Nutrition Surveys
SD: Standard Deviations
SMART: Standardized Monitoring and Assessment of Relief and Transitions
WHO: World Health Organization
