# Supplementary material for: An uncertainty estimate of the prevalence of stunting in national surveys: the need for better precision
Source: BMC Public Health. 2020 Nov 1;20:1634. doi: 10.1186/s12889-020-09753-8 (PMC7603753; doi:10.1186/s12889-020-09753-8)
Supplement: Supplementary file 1 — Additional file 1. National Family Health Survey-4 (NFHS-4) methodology; This file provides details of the NFH 4 survey methodology [file 12889_2020_9753_MOESM1_ESM.docx]

**National Family Health Survey-4 (NFHS-4) methodology:**

The survey was carried out from January 2015 to December 2016 and covered both urban and rural areas from 640 districts of India, of 29 states and 7 union territories (1). The height measurements were obtained from a total of 259 627 children <5 y of age, from 180 227 households with 237 136 valid height measurements (outliers excluded), using standard procedures and equipment. An infantometer or stadiometer (Seca 417 and 213) was used to measure recumbent length and standing height of children <2 and >2 y of age respectively (1). Households were given scores based on the number and kinds of consumer goods owned, and housing characteristics such as source of drinking water, toilet facilities, and flooring materials. National wealth quintiles were compiled by assigning the household score to each household member, ranking each person in the household population by their score, and then dividing the distribution into quintiles.

**Demographic Health Survey (DHS) methodology:**

The DHS are nationally representative household sample surveys in more than 85 LMICs, following standardised and representative sampling of participants with objective measurements of anthropometry (2). The latest round of DHS data with children < 5 y from 83 countries were downloaded (3).

**Computation of dispersion correction factor for DHS countries:**

The DHS survey data from 67 countries were selected to identify test datasets with characteristics matching the WHO MGRS sample as described for NFHS-4 (1). Indian data in the DHS were reported from the NFHS-4 (1). In addition, 17 countries had adequate data to provide a minimum sample size of 30 in the test dataset, and were chosen to calculate the dispersion correction factor for height-for-age. This sample size was sufficient to fulfil the

**Online Supplementary Material**

sample size requirement for the $\chi^{2}$test for the correction factor$\delta$, which was calculated after excluding the upper and lower 5 percentile values of height-for-age.

References:

1. International Institute of Population Science (IIPS). National Family Health Survey India (NFHS-4) 2015-2016 Report [Internet], 2016. Available from: <http://rchiips.org/nfhs/factsheet_nfhs-4.shtml> (Date accessed: 27 January, 2019).
2. Corsi DJ, Neuman M, Finlay JE, Subramanian SV. Demographic and health surveys: a profile. Int J Epidemiol 2012; 411602–1613.
3. The Demographic and Health Surveys Program DHS overview. Available from: <https://dhsprogram.com/what-we-do/survey-Types/dHs.cfm>. (Date accessed:  1 April, 2019)
