# Supplementary material for: An uncertainty estimate of the prevalence of stunting in national surveys: the need for better precision
Source: BMC Public Health. 2020 Nov 1;20:1634. doi: 10.1186/s12889-020-09753-8 (PMC7603753; doi:10.1186/s12889-020-09753-8)
Supplement: Supplementary file 2 — Additional file 2. Derivation of test statistic based on Z-score; Describes the derivation of test statistic over or under dispersion [file 12889_2020_9753_MOESM2_ESM.docx]

**Derivation of test statistic based on Z-score:**

The statistical test was constructed based on the Z-score derived by the WHO standard for height-for-age in the test dataset because of the following justification.

The Z-score by WHO standard was defined as

$$Z_{i}=\frac{Y_{ti}-\mu_{t}^{WHO}}{\sigma_{t}^{WHO}}$$

Applying the Jacobian transformation (1), it can be shown that the Z-score would be normally distributed with mean ‘0’ and variance $\delta^{2}$ based on our assumption overdispersion. That is $Z_{i}\sim N(0,\delta^{2})$. Hence the conventional test statistic for the above mentioned hypothesis would be as follows (2)

$$\chi_{stat}^{2}=\frac{ns_{z}^{2}}{\delta^{2}}\sim\chi_{n}^{2}$$

The test statistic should have $\chi^{2}$ distribution with ‘n’ degrees of freedom as mean of Z-score was assumed to be zero. Under the Null hypothesis ($H_{0}:\delta=1$) the test statistic reduced to

$$\chi_{stat}^{2}=ns_{z}^{2}=\sum_{i=1}^{n} Z_{i}^{2}$$

**References**

1. Mathai AM. Jacobians of matrix transformations and functions of matrix arguments. World Scientific Publishing Company, 1997.
2. Rao, CR. Linear Statistical Inference and its Applications. 2^nd^ edition. JWC Inc, 1976
