# Supplementary material for: An uncertainty estimate of the prevalence of stunting in national surveys: the need for better precision
Source: BMC Public Health. 2020 Nov 1;20:1634. doi: 10.1186/s12889-020-09753-8 (PMC7603753; doi:10.1186/s12889-020-09753-8)
Supplement: Supplementary file 3 — Additional file 3: Additional Figure 1. Flow diagram of country selection from DHS data to calculate the prevalence of relative stunting; DHS: Demographic Health Survey [file 12889_2020_9753_MOESM3_ESM.docx]

**ADDITIONAL FIGURE 1: Flow diagram of country selection from DHS data to calculate the prevalence of relative stunting; DHS: Demographic Health Survey (8)**

DHS data from 83 countries with children <5 y

Excluded data of 16 countries

-No data on anthropometry indices

-Inadequate socio-demographic data

Selected DHS data of 17 countries to identify test datasets with comparable characteristics to WHO MGRS sample; and calculate statistical correction factor for over dispersion ($\delta$)

Selected DHS data of 11 countries to calculate relative stunting

DHS data on height-for-age in children <5 y from 67 countries

Excluded data of 50 countries

-Inadequate matching variables: urban locality, socio-economic status, mother’s education, non-smoking mothers, exclusive breast feeding for 4 months, partial breast feeding for 12 months

-Survey sample size <30

Excluded data of 6 countries where $\delta$ was non-significant for $\chi^{2}$ test
