# Supplementary material for: An uncertainty estimate of the prevalence of stunting in national surveys: the need for better precision
Source: BMC Public Health. 2020 Nov 1;20:1634. doi: 10.1186/s12889-020-09753-8 (PMC7603753; doi:10.1186/s12889-020-09753-8)
Supplement: Supplementary file 4 — Additional file 4: Additional Figure 2. Distribution of height-for-age in NFHS-3 and MGRS [file 12889_2020_9753_MOESM4_ESM.docx]

**ADDITIONAL FIGURE 2**: **Distribution of height-for-age in NFHS-3 and MGRS;**

(A) Median and 95% CI of height-for-age from MGRS and NFHS-3 (B) Median and 95% CI of height-for-age from MGRS and NFHS- 3 test dataset; MGRS (n= 8440); NFHS-3 dataset (n= 43 753); NFHS-3 test dataset (n= 873). CI: Confidence Interval; MGRS: WHO Multicentre Growth Reference Study (5); NFHS-3: National Family Health Survey-3 (12)

**
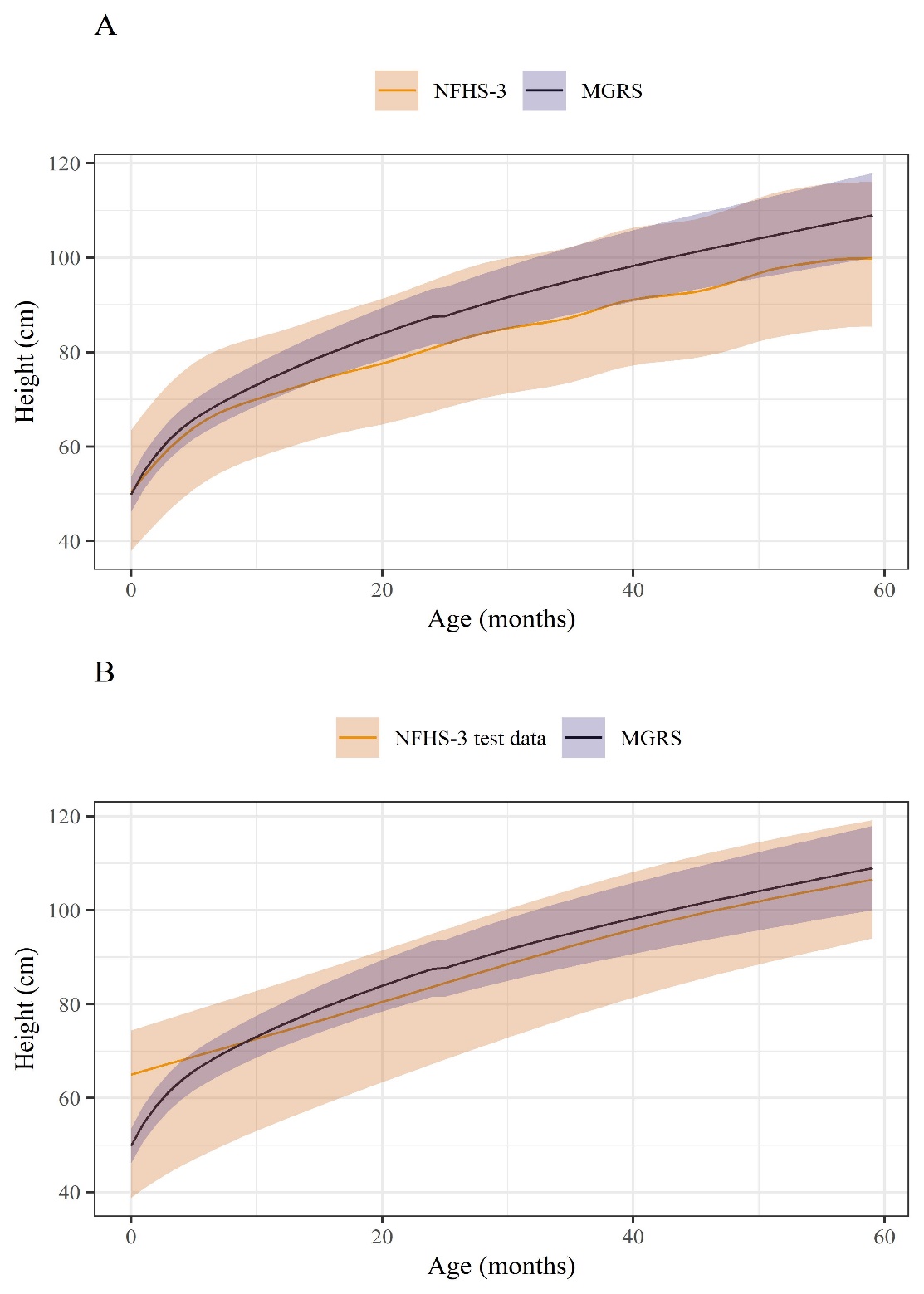
**
