# Supplementary material for: An uncertainty estimate of the prevalence of stunting in national surveys: the need for better precision
Source: BMC Public Health. 2020 Nov 1;20:1634. doi: 10.1186/s12889-020-09753-8 (PMC7603753; doi:10.1186/s12889-020-09753-8)
Supplement: Supplementary file 5 — Additional file 5: Additional Figure 3. Distribution of height-for-age in NFHS-4 test data set and MGRS [file 12889_2020_9753_MOESM5_ESM.docx]

**ADDITIONAL FIGURE 3**: **Distribution of height-for-age in NFHS-4 test data set and MGRS;**

Median and 95% CI of height-for-age from MGRS and NFHS-4 test dataset; MGRS (n= 8440); NFHS-4 test dataset (n= 3732); CI: Confidence Interval; MGRS: WHO Multicentre Growth Reference Study (5); NFHS-4: National Family Health Survey-4 (4)

**
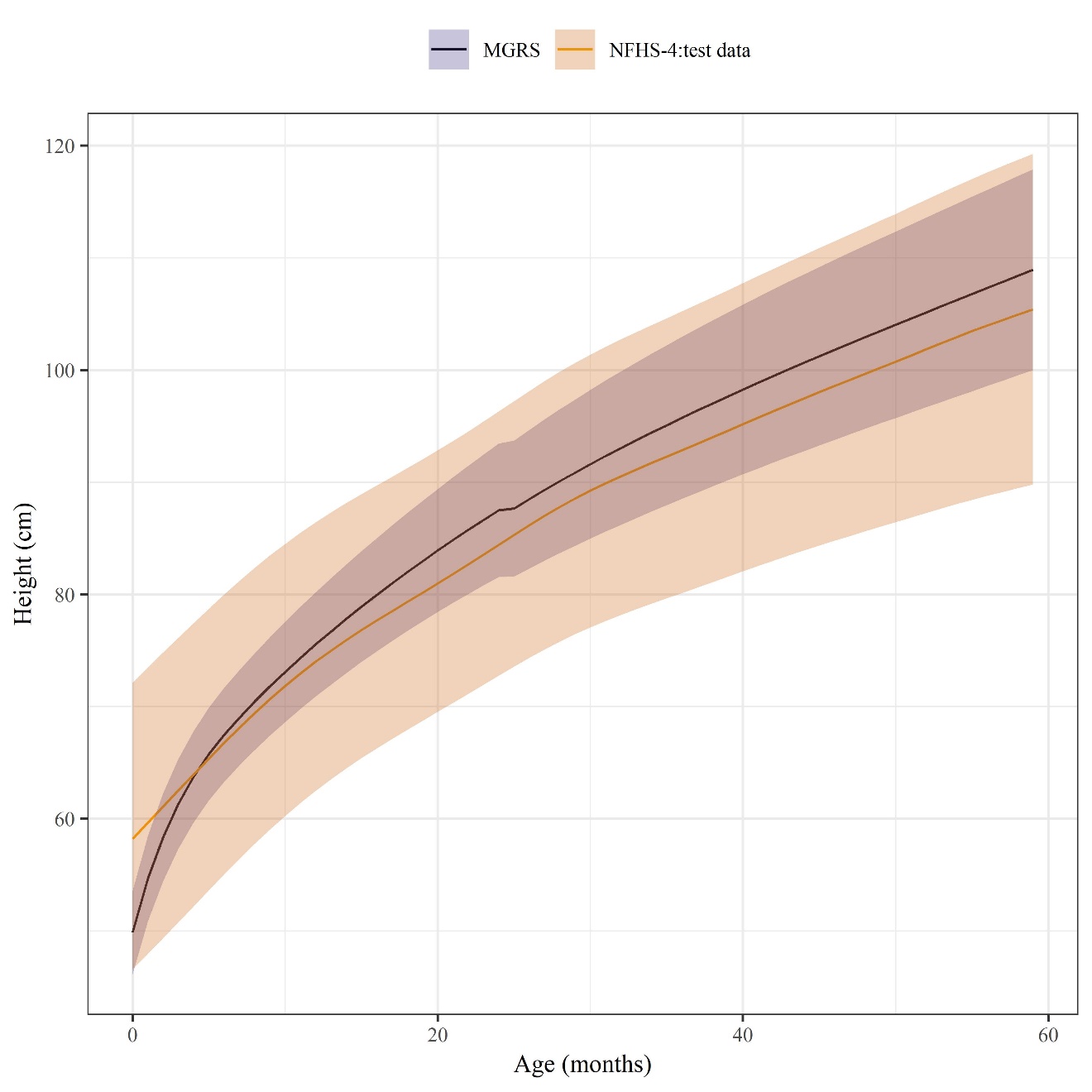
**
